# Supplementary material for: Maize GO Annotation—Methods, Evaluation, and Review (maize‐GAMER)
Source: Plant Direct. 2018 Apr 11;2(4):e00052. doi: 10.1002/pld3.52 (PMC6508527; doi:10.1002/pld3.52)
Supplement: Supplementary file 1 [file PLD3-2-e00052-s001.docx]

Maize GO Annotation - Methods, Evaluation, and Review (maize-GAMER)

Supplementary Information

## Kokulapalan Wimalanathan1,2, Iddo Friedberg1,3, Carson M. Andorf 4,5, and Carolyn J. Lawrence-Dill1,2,6,*

1Bioinformatics and Computational Biology, Iowa State University, Ames, IA 50011, USA

2Department of Genetics Development and Cell Biology, Iowa State

University, Ames, IA 50011, USA

3Department of Veterinary Microbiology and Preventive Medicine, Iowa State University, Ames, IA 50011, USA

4USDA-ARS Corn Insects and Crop Genetics Research Unit, Iowa State

University, Ames, IA 50011, USA

5Department of Computer Science, Iowa State University, Ames, IA 50011, USA

6Department of Agronomy, Iowa State University, Ames, IA 50011, USA

*Correspondence to [triffid@iastate.edu](mailto:triffid@iastate.edu)

# Supplementary Materials

## Supplementary Tables referenced in the main text.

**Table S1:** Top 10 plants by number of high-confidence GO annotations in UniProt-GOA

| **Rank** | **Species** | **Proteins** | **Annotations** |
| --- | --- | --- | --- |
| 1 | *Arabidopsis thaliana* | 3*,*702 | 72*,*089 |
| 2 | *Glycine max* | 3*,*847 | 43*,*202 |
| 3 | *Oryza sativa (Japonica Group)* | 39*,*947 | 32*,*750 |
| 4 | *Populus trichocarpa* | 3*,*694 | 31*,*851 |
| 5 | *Solanum lycopersicum* | 4*,*081 | 24*,*250 |
| 6 | *Sorghum bicolor* | 4*,*558 | 23*,*470 |
| 7 | *Vitis vinifera* | 29*,*760 | 23*,*350 |
| 8 | *Brachypodium distachyon* | 15*,*368 | 22*,*454 |
| 9 | *Physcomitrella patens* | 3*,*218 | 18*,*348 |

**Table S2:** *hFmax* and score thresholds for mixed-method pipelines by GO categories

| Cellular Component | | |
| --- | --- | --- |
| Pipeline | *hFmax* | Pipeline Threshold |
| Argot2 | 0*.*572 | 0*.*05 |
| FANN-GO | NA | NA |
| PANNZER | 0*.*460 | 0*.*20 |
|  | | |
| Molecular Function | | |
| Pipeline | *hFmax* | Pipeline Threshold |
| Argot2 | 0.584 | 0.15 |
| FANN-GO | 0.582 | 0.65 |
| PANNZER | 0.607 | 0.55 |
|  | | |
| Biological Process | | |
| Pipeline | *hFmax* | Pipeline Threshold |
| Argot2 | 0.300 | 0.15 |
| FANN-GO | 0.272 | 0.30 |
| PANNZER | 0.241 | 0.40 |

Mixed-method pipeline scores that result in the max *hF*1 calculated for each pipeline for each GO category. The threshold values shown here were used to select high confidence GO annotations from each pipeline and only annotations with a score ≥ threshold were selected. *It is important to note that absolute values from the pipelines were normalized between 0-1 before hF*1 *scores were calculated and hFmax was determined.* NA indicates that the pipeline did not annotate terms in the GO category.

# Supplementary Figures

#
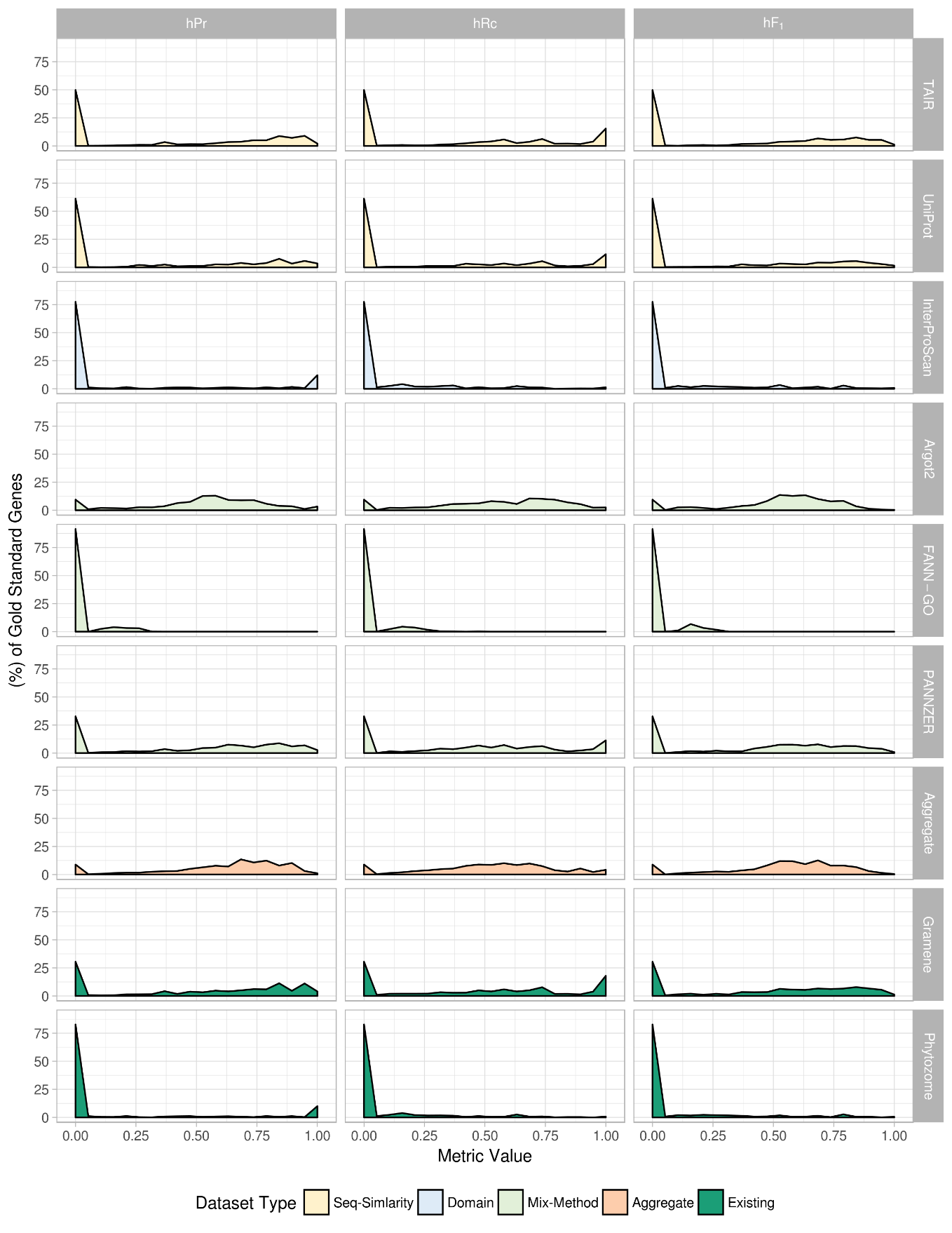


Figure S 1: Distribution of evaluation metrics for Gold Standard Genes

Distribution of metrics hRc, hPr, and hF_1_ for each annotation method based on genes in the gold standard dataset. If a particular annotation method did not have an annotation for a gene in the gold standard, it was assigned a value of 0. The bin width used to calculate the distribution was 0.05.


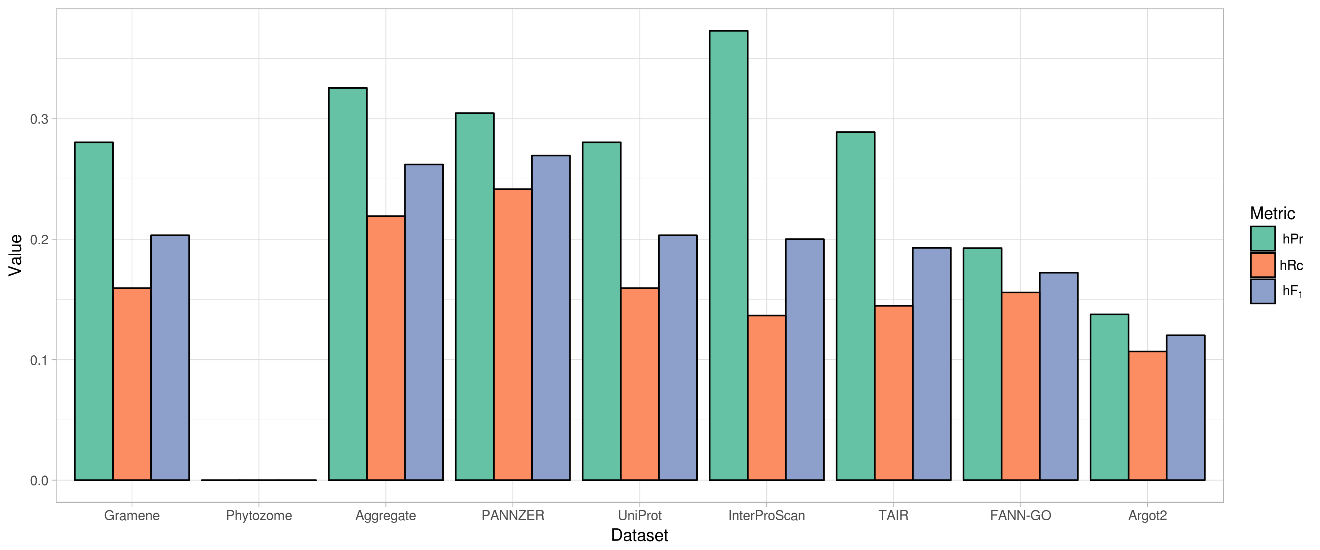


Figure S 2: Evaluation metrics calculated for na1 gene for each dataset

Metrics hPr, hRc, and hF1 for existing (Gramene and Phytozome) datasets as well as the maize-GAMER aggregate and component datasets. hPr, hRc, and hF_1_ are shown in green, orange, and periwinkle, respectively.

# Detailed Metrics

## Hierarchical Precision

For gene $g$ with annotation $AS_{i}$ in annotation set and annotation $GS_{j}$ in the gold standard the $hPr$ is calculated as follows

$$hPr_{(g,AS_{i},GS_{j})}=\frac{GO_{AS_{i}}\cap GO_{GS_{j}}}{GO_{AS_{i}}}$$

Where:

$GO_{AS_{i}}$: GO terms inferred for annotation $AS_{i}$ by propagating the GO hierarchy till the root term
$GO_{GS_{j}}$: GO terms inferred for annotation $GS_{j}$ by propagating the GO hierarchy root term

For a gene $g$ with annotation $AS_{i}$ in annotation set in the GO ontology $o$ the $hPr$ is calculated as followed.

$$hPr_{(g,AS_{i})}=\sum_{j\in GS_{g,o}} \frac{hPr_{(g,AS_{i},GS_{j})}}{|GS_{g,o}|}$$

Where:

$GS_{g,o}$ : GO terms annotated to gene $g$ in the ontology $o$ in gold standard $GS$

For gene $g$, $hPr$ for ontology $o$ is calculated as follows

$$hPr_{(g)}=\sum_{i\in AS_{g,o}} \frac{hPr_{(g,AS_{i})}}{|AS_{g,o}|}$$

Where:

$AS_{g,o}$: GO terms annotated to gene $g$ in the ontology $o$ in the annotation set $AS$.

Note: Only genes with GO terms annotated in ontology $o$ in both $AS$ and $GS$ can be used for this calculation.

$hPr$ for a given annotation set $AS$ for the ontology $o$ is calculated as followed

$$hPr_{(AS,o)}=\sum_{g\in(AS_{o}\cap GS_{o})} \frac{hPr_{(g)}}{|AS_{o}\cap GS_{o}|}$$

Where:

$AS_{o}$: Genes annotated in the annotation set $AS$ in the ontology $o$
$GS_{o}$: Genes annotated in the gold standard $GS$ in the ontology $o$

## Hierarchical Recall

For gene $g$ with annotation $AS_{i}$ in annotation set and annotation $GS_{j}$ in the gold standard the $hRc$ is calculated as follows

$$hRc_{(g,AS_{i},GS_{j})}=\frac{GO_{AS_{i}}\cap GO_{GS_{j}}}{GO_{GS_{i}}}$$

Where:

$GO_{AS_{i}}$: GO terms inferred for annotation $AS_{i}$ by propagating the GO hierarchy till the root term
$GO_{GS_{j}}$ & GO terms inferred for annotation $GS_{j}$ by propagating the GO hierarchy root term

For a gene $g$ with annotation $GS_{i}$ in gold standard in the GO ontology $o$ the $hRc$ is calculated as followed.

$$hRc_{(g,GS_{j})}=\sum_{i\in AS_{g,o}} \frac{hRc_{(g,AS_{i},GS_{j})}}{|AS_{g,o}|}$$

Where:

$AS_{g,o}$: GO terms annotated to gene $g$ in the ontology $o$ in annotation set $AS$

For gene $g$, $hRc$ for ontology $o$ is calculated as follows

$$hRc_{(g)}=\sum_{j\in GS_{g,o}} \frac{hRc_{(g,GS_{j})}}{|GS_{g,o}|}$$

Where:

$GS_{g,o}$: GO terms annotated to gene $g$ in the ontology $o$ in the gold standard $GS$.

Note: Only genes with GO terms annotated in ontology $o$ in both $AS$ and $GS$ can be used for this calculation.

$hRc$ for a given annotation set $AS$ for the ontology $o$ is calculated as followed

$$hRc_{(AS,o)}=\sum_{g\in(AS_{o}\cap GS_{o})} \frac{hPr_{(g)}}{|AS_{o}\cap GS_{o}|}$$

Where:

$AS_{o}$: Genes annotated in the annotation set $AS$ in the ontology $o$
$GS_{o}$: Genes annotated in the gold standard $GS$ in the ontology $o$

# Reproducibility

## Datasets

**Table 2.1:** Public datasets used in the project

| **Database** | **Type** | **Format** | **Version** | **Species** |
| --- | --- | --- | --- | --- |
| TAIR | Protein Sequences | fasta | TAIR 10 | *Arabidopsis thaliana* |
| TAIR | GO Annotations | gaf 2.0 | TAIR 10 (20170410) | *Arabidopsis thaliana* |
| Gramene 49 | Gene Annotations | gff3 | 5b+ | *Zea mays* |
| Gramene 49 | GO Annotations | gaf 2.0 | 5b+ | *Zea mays* |
| Phytozome 11 | GO Annotations | tsv | 5b+ | *Zea mays* |
| Uniprot | Protein sequences | fasta | 20170410 | All species |
| Uniprot | Protein sequences | fasta | 20170410 | All plants |
| Uniprot | GO Annotations | gaf 2.0 | 20170410 | All plants |
| Pfam | HMMs | hmm | 27.0 | All species |
| PANTHER | HMMs | hmm | 10.0 | All species |

## Software Tools and Versions

**Table 2.2:** Software tools used in the project. Exact parameters used are specified in section 2.3.

| **Software** | **Type** | **Version** | **Citation** |
| --- | --- | --- | --- |
| NCBI-BLAST | Sequence similarity | 2.6.0 | (Altschul et al., 1990) |
| HMMER | HMM scanning | 3.1b1 | (Finn et al., 2011) |
| InterProScan | GO Annotation | 5.15-55.0 | (Jones et al., 2014) |
| PANNZER | GO Annotation | 1.1 | (Koskinen et al., 2015) |
| Argot2 | GO Annotation | 2.0 (Web Server) | (Falda et al., 2012) |
| FANN-GO | GO Annotation | One version only | (Clark and Radivojac, 2011) |
| AIGO | GO Evaluations | 0.1.0 | (Defoin-Platel et al., 2011) |
| FASTX-Toolkit | Fasta manipulation | 0.0.13 | (Gordon and Hannon, 2010) |

## Annotation of Maize Genes

### Obtaining Input Datasets

1. Query Sequences
   1. Downloaded maize RefGen_v3 5b+ protein sequences as a fasta file from Gramene
   2. Longest translated protein sequence among the transcript models for each gene was retained to represent a gene
   3. Transcript model id was renamed to the gene model ID and generate a maize-filtered fasta file
   4. Made a BLAST database using the maize-filtered fasta file
   - makeblastdb -in ‘maize-filtered.fa'
      -dbtype ‘prot'
      -hash_index
      -out ‘maize-filtered'
      -title ‘maize-filtered'
2. *Arabidopsis*
   1. Downloaded Arabidopsis transcript protein sequences from TAIR v10
   2. Made an arabidopsis-filtered fasta by filtering for the transcript model with the longest protein sequence to represent each gene and rename transcript model IDs to gene model IDs
   3. Made a blast database using the arabidopsis-filtered fasta file
   - makeblastdb -in ‘arabidopsis-filtered.fa'
      -dbtype ‘prot'
      -hash_index
      -out ‘arabidopsis-filtered'
      -title ‘arabidopsis-filtered'
   1. Downloaded the *Arabidopsis* GO annotation GAF file arabidopsis.gaf from TAIR v10
   2. Filter the GO annotations from arabidopsis.gaf to retain the annotations which have curated evidence codes and convert to a GAF file named filtered-arabidpsis.gaf
      1. Selected [EXP, IDA, IPI, IMP, IGI, IEP, ISS, ISO, ISA, ISM, IGC, IBA, IBD, IKR, IRD, RCA, TAS, IC]
      2. Omitted [IEA, ND, NAS]
3. UniProt Plants
   1. Curated GO annotations for plants were downloaded from UniProt using QuickGO
   - wget -O annot/tmp.gaf
      "http://www.ebi.ac.uk/QuickGO/GAnnotation?\
     - format=gaf&limit=-1&q=!evidence=IEA,ND,NAS&tax=33090"
   1. GO annotations from Top 10 plant species with highest number of annotations from tmp.gaf were filter and saved to uniprot-hc-plant.gaf
   2. NCBI taxonomy IDs of the top 10 plant species at the time of downloading UniProt data are
      (15368, 29760, 3055, 3218, 3694, 3702, 3847, 39947, 4081, 4558)
   3. Plant Protein sequences with curated GO annotations were downloaded from UniProt using QuickGO tool
   - wget -O fa/tmp.fa
      "http://www.ebi.ac.uk/QuickGO/GAnnotation?\
     - format=fasta&limit=-1&q=!evidence=IEA,ND,NAS&tax=33090"
   1. Convert the IDs in tmp.fa to match the protein IDs in the uniprot-hc-plant.gaf file and make a new fasta file tmp2.fa
   2. tmp2.fa was filtered for IDs present in the uniprot-hc-plant.gaf and save to uniprot-hc-plant.fa
   3. Make a BLAST database for the uniprot-hc-plant.fa
   - makeblastdb -in ‘uniprot-hc-plant.fa'
      -dbtype ‘prot'
      -out ‘uniprot-hc-plant'

### Sequence-Similarity methods

1. *Arabidopsis*
   1. Used the maize-filtered fasta as query and searched against uniprot-hc-plant blast database
   - blastp -db ‘arabidopsis-filtered'
      -query ‘maize-filtered.fa'
      -max_target_seqs ‘20'
      -out ‘mz-ara-aa.txt'
      -outfmt ‘6 qseqid sseqid qlen qstart qend slen sstart send
     - - evalue bitscore score length pident nident gaps'

-num_threads ‘16'

- 1. Used the arabidopsis-filtered fasta as query and searched against maize-filtered database
  - blastp -db ‘maize-filtered'
     -query ‘arabidopsis-filtered.fa'
     -max_target_seqs ‘20'
     -out ‘ara-mz-aa.txt'
     -outfmt ‘6 qseqid sseqid qlen qstart qend slen sstart send
    - - evalue bitscore score length pident nident gaps'
    - -num_threads ‘16'
  1. Used custom R script to read ‘ara-mz-aa.txt’ & ‘mz-ara-aa.txt’ and obtained Reciprocal-Best-Hits (RBH) saved as ‘maize-v3-vs-tair10.rbh.txt’
     Specific parameters used within the script
     - BLAST hits were filtered by using an e-value cut off of 10e-10 form both datasets
     - Hits were ranked by the score in descending order
  2. Used custom R script to read ‘filtered-arabidpsis.gaf’ and maize-v3-vs-tair10.rbh.txt inherited the curated GO terms from *Arabidopsis* to maize and created a ‘maize-arabidopsis.gaf’ file

1. UniProt Plants
   1. Used the maize-filtered fasta as query and searched against uniprot-hc-plant database
   - blastp -db ‘uniprot-hc-plant'
      -query ‘maize-filtered.fa'
      -max_target_seqs ‘20'
      -out ‘mz-uniprot-aa.txt'
      -outfmt ‘6 qseqid sseqid qlen qstart qend slen sstart send
     - - evalue bitscore score length pident nident gaps'

-num_threads ‘16'

- 1. Used the uniprot-hc-plant fasta as query and searched against maize-filtered database
  - blastp -db ‘maize-filtered'
     -query ‘uniprot-hc-plant.fa'
     -max_target_seqs ‘20'
     -out ‘uniprot-mz-aa.txt'
     -outfmt ‘6 qseqid sseqid qlen qstart qend slen sstart send
    - - evalue bitscore score length pident nident gaps'

-num_threads ‘16'

- 1. Used custom R script to read ‘uniprot-mz-aa.txt’ & ‘mz-uniprot-aa.txt’ and obtained Reciprocal-Best-Hits (RBH) saved as ‘maize-v3-vs-uniprot.rbh.txt’
     Specific steps and parameters used within the script
     - BLAST hits were filtered by using an e-value cut off of 10e-10 form both datasets
     - Hits were ranked by the score in descending order
     - RBH assignment was performed for each of the 10 plant species in uniprot-hc-plant.gaf and maize the steps were exactly similar to *Arabidopsis*
  2. Used custom R script to read ‘uniprot-hc-plant.gaf’ and maize-v3-vs-uniprot.rbh.txt inherited the curated GO terms from Plants in Uniprot dataset to maize and created a ‘maize-uniprot.gaf’ file

### Domain Prescence method

1. InterProScan5 pipeline was downloaded an configured in a local server
2. Necessary PANTHER database was downloaded added to the data location
3. InterProScan5 pipeline was run on maize-filtered.fa file to assign putative domains and assign GO terms to genes

- interproscan.sh -i ‘maize-filtered.fa'
   -goterms
   -f ‘tsv'
   -o ‘maize-filtered.iprs.out'

1. A custom Rscript was used to convert ‘maize-filtered.iprs.out’ to ‘maize-interproscan.gaf’

### Mixed-method pipelines

1. Common Pre-processing steps
   1. All protein sequences was downloaded from UniProt as uniprot.fa
   2. The uniprot.fa was used to make a blast database
   - makeblastdb -in ‘uniprot.fa'
      -dbtype ‘prot'
      -out ‘uniprot'
   1. Pfam-A and Pfam-B HMM models were downloaded and uncompressed
   2. A Pfam-AB file was created by concatenating Pfam-A and Pfam-B
   - cat "Pfam-A.hmm" and "Pfam-B.hmm" > "Pfam-AB.hmm"
     hmmpress "Pfam-AB.hmm"
2. Argot2
   1. “maize-filtered.fa” was split into smaller files with only 5000 sequences per file. Argot2 webserver allows a max 5000 sequences to be batch processed at a time.
   2. BLASTP was run against the UniProt database for each split file
   - blastp -outfmt ‘6 qseqid sseqid evalue'
      -num_threads ‘16'
      -query ‘maize-filtered.1.fa'
      -db ‘uniprot'
      -out ‘maize-filtered.1.blast'
   1. Hmmer was used for each split file to search against the Pfam HMMs
   - hmmscan --cpu ‘16'
      --tblout ‘maize-filtered.1.hmmer'
      ‘Pfam-AB.hmm'
      ‘maize-filtered.1.fa'
   1. All output files were compressed as zip filess
   2. Output files from BLAST and Hmmer for each split fasta file was submitted as a new job for batch processing on the Argot2 website (http://www.medcomp.medicina.unipd.it/Argot2/)
   3. Argot2 Results for each part was downloaded and renamed according to input file names
   4. Argot2 results were converted to GAF 2.0 format using an Rscript
   5. This was saved as ‘maize-argot2.gaf’ file
3. PANNZER
   1. PANNZER files and database were downloaded from <http://ekhidna.biocenter.helsinki.fi/pannzer/Download.html>
   2. PANNZER tool was setup according to the instructions provided in the manual
   3. BLASTP was used to query the maize-filtered split fasta files used for Argot2 against the uniprot blast database
   - blastp -db ‘uniprot'
      -query ‘maize-filtered.1.fa'
      -outfmt ‘5'
      -num_threads ‘32'
      -out ‘maize-filtered.1.xml'
   1. A config file required for PANNZER for each BLAST xml file output in the previous step
   - [GENERAL_SETTINGS]
     INPUT_TYPE=BLASTXML
     INPUT_FILE=maize-filtered.1.xml
     XML=True
     DATA_FOLDER=PANNZER/db/
     DB=uniprot
     RESULT_FOLDER=ouput/
     RESULT_BASE_NAME=maize-filtered.1
     INPUT_BASE_NAME=Prefix_of_the_desc_file
     INPUT_FOLDER=xml/
     QUERY_TAXON=4577
     GET_TAXON=False
     GENERATE_IDF=False
     MULTIPLE_SPECIES=False

     [TRESHOLD_VALUES]
     BITSCORE=50
     SEQUENCE_LENGTH=20
     IDENTITY_PERCENT=50
     E-VALUE=0
     TARGET_COVERAGE=0.6
     QUERY_COVERAGE=0.6
     INFORMATIVE=30
     INFORMATIVE_HITS=100
     CLUSTER=0.3

     [MYSQL]
     SQL_DB_HOST=localhost
     SQL_DB_PORT =
     SQL_DB_USER = pannzer
     SQL_DB_PASSWORD = pannzer
     SQL_DB = pannzer

     [TAXONOMY]
     DB=taxonomy-all.tab
     CALCULATE=True
     NODE_SELECTOR=1
     TRACK_GROUPS=False
     TRACKED_GROUPS=
     ONLY_ONE_HIT_PER_SPECIE=False

     [GO]
     WRITE_GO=True
     OBO=gene_ontology_ext.obo
     ID_MAPPING=idmapping_selected.tab
     ENZYME=enzyme.dat

     [LEVEL_OF_PRINTING]
     SIMPLE_OUTPUT=True
     CLUSTER=True
     CLUSTER_MEMBERS=False
     ALL=False
     ERROR=True
     DEBUG=False
     INFO=False

     [EVALUATION] ### IF YOU DON'T KNOW WHAT YOU ARE DOING, DON'T CHANGE ANYTHING FROM THIS ON!!!!
     PRINT_EVAL=False
     TEST=False
     OTHER=False
   1. PANNZER was run for each config file
   2. All results files from each split fasta files were concatenated and converted to a GAF 2.0 file
   3. GAF file was saved as ‘maize-pannzer.gaf’
4. FANN-GO
   1. FANN-GO tool was downloaded from <http://montana.informatics.indiana.edu/fanngo/fanngo.html>, uncompressed and installed.
   2. The “maize-filtered.fa” file was used as input and FANN-GO was run using the following code
   - echo off all
     cd code
     [Headers, Sequences] = fastaread('maize-filtered.fa')
     PRED=MAIN(Sequences)
     Headers = transpose(Headers)
     Headers = regexprep(Headers, ‘ .*', ‘')
     tbnames = horzcat('gene_id',PRED.accessions)
     tbnames = strrep(tbnames,':','_')
     scores = num2cell(PRED.scores)
     all_scores = horzcat(Headers,scores)
     all_scores = cell2table(all_scores)
     all_scores.Properties.VariableNames = tbnames
     writetable(all_scores,'../scores.txt','Delimiter','\t')
   1. “scores.txt” was converted into a GAF 2.0 file and saved as ‘maize-fanngo.gaf’

## Selection and Cleaning of Maize Annotations

### Selection of High-Confidence Mixed-Method Annotations

1. Score thresholds determined for the mixed-method pipeline annotations by evaluation against the MaizeGDB gold standard dataset. (Please refer to the main paper for how the score thresholds were determined)
   1. Argot2
      1. BP : 0.15
      2. CC : 0.05
      3. MF : 0.15
   2. FANN-GO
      1. BP : 0.3
      2. MF : 0.65
   3. PANNZER
      1. BP : 0.4
      2. CC : 0.2
      3. MF : 0.55
2. ’maize-argot2.gaf’, ‘maize-fanngo.gaf’ and ‘maize-pannzer.gaf’ files were filtered to retain annotations with a score greater than or equal to the thresholds mentioned in the previous step

### List of Component Datasets

1. Sequence-Similarity
   1. maize-arabidopsis.gaf
   2. maize-uniprot.gaf
2. Domain-Presence
   1. maize-interproscan.gaf
3. Mixed-method Pipelines
   1. maize-argot2.gaf
   2. maize-pannzer.gaf
   3. maize-fanngo.gaf

### Cleaning Duplications

1. Datasets mentioned in section 4.4.2 were cleaned for duplicate annotations
2. Duplicate annotation is when the same GO term is annotated to the same gene more than one time
3. Duplicate annotations were replaced by a single instance of the Gene-GO term pair independently for each dataset
4. The unique annotations for each component dataset was saved as gaf file

### Cleaning Redundancy

1. Redundancy is the annotation of one or more ancestral GO terms to a gene which is annotated to a more specific offspring GO term
2. Unique datasets mentioned in section 4.4.3 were cleaned by removing annotations with redundant GO terms
3. The non-redundant annotations for each component dataset was saved as gaf file

### Making Aggregate Dataset

1. All the non-redundant component datasets from section 4.4.4 were concatenated together
2. The duplication introduced by merging multiple component datasets was cleaned using the steps from section 4.4.3
3. The redundancy introduced by merging multiple component datasets was cleaned using the steps from section 4.4.4
4. The non-redundant aggregate dataset was saved as ‘maize-aggregate.gaf’

# References

Altschul, S.F., Gish, W., Miller, W., Myers, E.W., and Lipman, D.J. (1990). Basic local alignment search tool. J Mol Biol *215*, 403–410.

Clark, W.T., and Radivojac, P. (2011). Analysis of protein function and its prediction from amino acid sequence. Proteins *79*, 2086–2096.

Defoin-Platel, M., Hindle, M.M., Lysenko, A., Powers, S.J., Habash, D.Z., Rawlings, C.J., and Saqi, M. (2011). AIGO: towards a unified framework for the analysis and the inter-comparison of GO functional annotations. BMC Bioinformatics *12*, 431.

Falda, M., Toppo, S., Pescarolo, A., Lavezzo, E., Di Camillo, B., Facchinetti, A., Cilia, E., Velasco, R., and Fontana, P. (2012). Argot2: a large scale function prediction tool relying on semantic similarity of weighted Gene Ontology terms. BMC Bioinformatics *13 Suppl 4*, S14.

Finn, R.D., Clements, J., and Eddy, S.R. (2011). HMMER web server: interactive sequence similarity searching. Nucleic Acids Res *39*, W29–37.

Gordon, A., and Hannon, G. (2010). Fastx-toolkit. FASTQ/A Short-reads Pre-processing Tools (unpublished) http://hannonlab. Cshl. Edu/fastx_toolkit.

Jones, P., Binns, D., Chang, H.-Y., Fraser, M., Li, W., McAnulla, C., McWilliam, H., Maslen, J., Mitchell, A., Nuka, G., et al. (2014). InterProScan 5: genome-scale protein function classification. Bioinformatics *30*, 1236–1240.

Koskinen, P., Törönen, P., Nokso-Koivisto, J., and Holm, L. (2015). PANNZER: high-throughput functional annotation of uncharacterized proteins in an error-prone environment. Bioinformatics *31*, 1544–1552.
