# Supplementary material for: Maize GO Annotation—Methods, Evaluation, and Review (maize‐GAMER)
Source: Plant Direct. 2018 Apr 11;2(4):e00052. doi: 10.1002/pld3.52 (PMC6508527; doi:10.1002/pld3.52)
Supplement: Supplementary file 2 [file PLD3-2-e00052-s002.pdf]

Response to reviewers:

Reviewer comments in black

Responses in blue

Reviewer #1:

The manuscript titled "Maize GO Annotation - Methods, Evaluation, and Review (maize-GAMER)", built a new complete annotation of maize protein coding genes, which is with much better performance than the available other databases, including Phytozome and Gramene. I highly appreciated this work, because it will be very helpful for maize researchers.

However, the authors claimed 100% coverage of all protein coding genes, and I am concerned that there will be many inaccurate annotations. Although the "gold standard" gene sets are shown more accurate, I don't understand why all the other genes could be annotated, as so many genes' functions are still unknown. One reason may be that compared to the other old annotation, this is a new analysis when more genes' functions have been studied recently, and the author's mixed method may also contribute to collecting more data. But I still doubt all the genes can be annotated, even with these two above reasons. I am afraid many wrong annotations exist in this "maize-GAMER". The authors mentioned it, "Mixed-method pipelines do have a limitation: they have higher coverage but annotations are less specific in general when compared with datasets produced using other approaches." Is it true that there are many wrong annotations?

Like the reviewer, we were concerned that creating a high-coverage dataset of predicted annotations would generate a number of annotations that were unreliable. To determine whether that was the case, we assessed existing predicted functional annotation datasets (i.e., the Gramene and Phytozome datasets) as well as the new maize-GAMER dataset based on concurrence with curated (rather than predicted) functional annotations (based on F-measure). These assessments demonstrate that the maize-GAMER predicted annotations are no more unreliable overall than other existing annotations on average. Beyond the fact that more genes gain annotations and more terms are annotated to each gene on average, the new maize-GAMER prediction set is documented at a level that a researcher could reproduce the result and can determine which of the methodologies resulted in each annotation in the aggregate dataset (i.e., InterproScan, RBH, FANN-GO, etc.). Because these annotations are specific in how they were created and are reproducible, we take the perspective that sharing access to these datasets not only expands the maize annotation set into a useful direction, it is an example of best practices for annotating a prediction such that others could decide for themselves the degree to which the result is useful for formulating hypotheses for their own research.

We have briefly discussed the issue in the discussion section to reiterate the value maize-GAMER dataset brings to the maize community. We have added text to this effect in the discussion section paragraph 4.

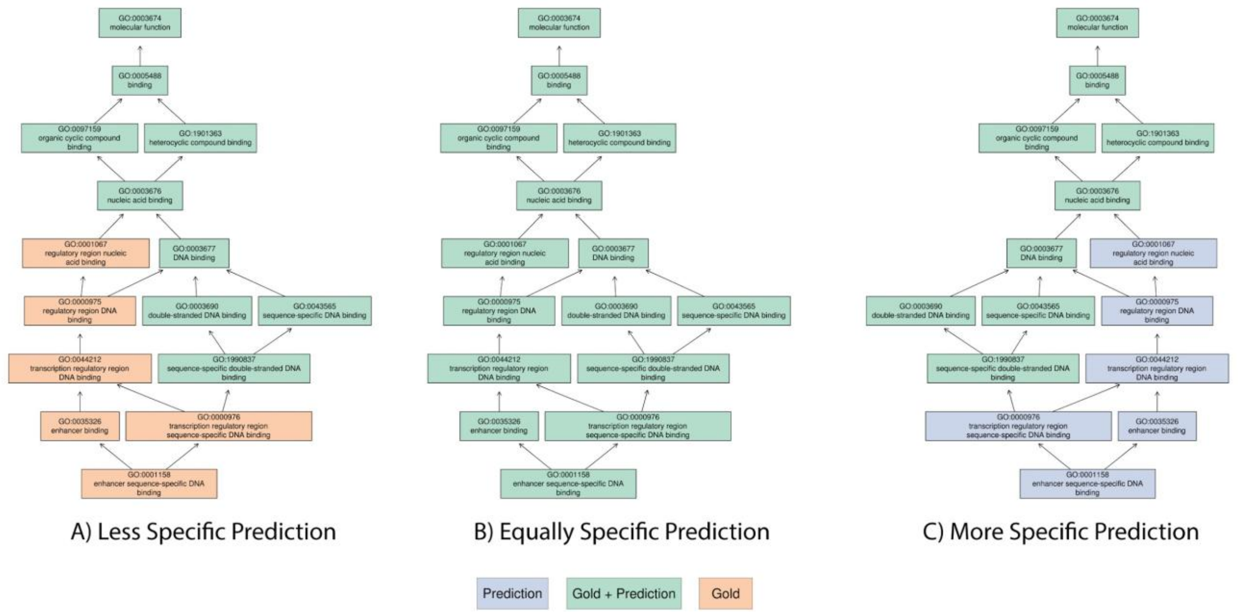

We have generated this illustration to clarify the details about specificity of the predicted GO annotations. Specificity of the predicted annotation can be classified into three categories. Case (A) where the prediction is less specific than the gold standard annotation, case (B) prediction and gold standard have same specificity, and case (C) where prediction is more specific than gold standard. Less specificity in the case of prediction is not always incorrect, but is not as detailed (granular) as the gold standard. This is a known issue when predicting GO terms, because the amount of data available are inadequate for the construction of mathematical or machine learning methods.

I am wondering whether the authors' efforts to annotate maize genes can be implemented in other plants, even animal. If so, can we still get 100% annotation rate?
Because we have made the code and methods available so that others could try out the methods, we are hopeful that these questions will be approached, by our own group and/or others. Preliminary analyses (not reported here) indicate that for other plants the system works well in other plants.

Table 2, "Descripti" should be "Description".
Thank you for bringing that to our notice. There indeed was a margin issue with the row and we have fixed it to show the full text of that cell.

Reviewer #3:
The manuscript entitled "Maize GO Annotation - Methods, Evaluation, and Review (maize-GAMER) " by Wimalanthan et al. report a new maize functional annotation resource that will be very helpful for the maize community and bioinformatics tools for gene functional annotation that can be applied to a wide range of organisms as well.

A major strength of this work is the use of a set of expertized annotation to guide the automatic annotation process. In addition, the pipeline is based on state of the art methods. The result is functional annotation of maize genes of an unprecedented richness and coverage. Without any doubt, this work will be very useful in many research interested in gene function.

Major comments
Maize-GAMER is an aggregate of many Component Annotation Sets. Some of these sets are made from distinct reference organisms. It is therefore possible that these components include conflicting or inconsistent annotation for a single gene. The hF_1 metrics can tell how far a predicted annotation is from the gold standard (GS) but no why it is. The annotation of a single gene may include more terms than the GS with the possibility of inconsistencies making the annotation of the gene difficult to use (decreased specificity ?). Or, in contrast, the predicted annotation may miss terms present in the gold standard. Or both. It may be interesting to discuss about this point. I would also appreciate to have a look at the hFf_1 and specificity distribution (for all gene of the GS) for each component and the aggregated annotation, it might be interesting and better support the conclusion of the na1 case study (part 4.3).

We thank the reviewer for the insightful comments. We have used the hF_1 metric to assess how the different methods used to produce the GO dataset for maize performed. The hF_1 metric is the harmonic mean of hierarchical Precision (hPr) and hierarchical Recall (hRc). These metrics are similar to Precision and Recall, but take the hierarchical nature of Gene Ontology into account when evaluating against the gold standard. hPr metric indicates the proportion of the GO terms predicted by the method that was correct, and a low value would reflect the inconsistencies when large number of GO terms are annotated by a method. This could be used to find how specific the annotation from a given method is. hRc gives the proportion of the GO terms in the gold standard dataset which are correctly predicted. Low hRc values would reflect the inability of a particular method to capture the GO terms annotated to a particular gene in the gold standard. We do agree the comparison of the distributions of hF1, hPr, and hRc would provide a clear picture of the value of the annotations from different component methods and other existing maize annotation datasets. We have provided **supplemental figure S1** with the distribution of hF1,hPr, and hRc from different component methods, aggregate maize-GAMER dataset and other existing maize GO datasets. We have added text in the discussion section to explain the differences among different tools.

Minor comments
3.5.1 "Redundancy is the proportion of shared ancestral terms annotated for each gene averaged across all genes."

While the term ancestral is well defined in Defoin-Platel et al. this term should be explained in this manuscript as it might be misleading to geneticists and evolution researchers.
We have defined and explained the ancestral GO terms right before Redundancy is defined in the text.

3.5.1 "An ideally cleaned annotation set for a single gene would have no duplication, no redundancy, and high coverage and specificity. "

Not sure what coverage means at the scale of a single gene if I consider the definition of coverage given above : "Coverage is the proportion of genes that have at least one GO term assigned. "
We thank the reviewer for the attention to detail, and agree that coverage does not apply to a single gene and have updated the text with correct phrasing to describe an annotation set rather than the gene

4.4.1 "The IPRS annotation set had a lower number of annotations compared to the CAFA mixed-method pipelines, but covered more genes than methods."

Covered more genes than which methods ?
We appreciate the comment, and we have updated the sentence to correct the mistake. It should have been “sequence-similarity methods” instead of “methods”.
